# The Health of Arab Americans in the United States: An Updated Comprehensive Literature Review

**DOI:** 10.3389/fpubh.2018.00262

**Published:** 2018-09-11

**Authors:** Nadia N. Abuelezam, Abdulrahman M. El-Sayed, Sandro Galea

**Affiliations:** ^1^Boston College, William F. Connell School of Nursing, Chestnut Hill, MA, United States; ^2^Department of Health, Detroit, MI, United States; ^3^School of Public Health, Boston University, Boston, MA, United States

**Keywords:** health disparities, arab american, stress, diabetes, mental health, women's health, child health, infectious disease

## Abstract

**Background:** Arab Americans are a historically understudied minority group in the United States and their health needs and risks have been poorly documented. We aim to provide an updated comprehensive review of the literature on Arab American physical and mental health and provide suggestions for future work in this field.

**Methods:** A comprehensive review of the English language medical and public health literature published prior to 2017 identified through multiple database searches was conducted with search terms describing Arab Americans and health outcomes and behaviors. The literature was qualitatively summarized by health behavior (vaccination, tobacco use, drug and alcohol use, and physical activity), health outcome (diabetes, mental health, cardiovascular disease, cancer, women's, and child health), and populations at increased risk of poor health outcomes (adolescents and the elderly).

**Results:** The majority of studies identified exploring Arab American health have been published since 2009 with an increase in the number of longitudinal and intervention studies done with this population. The majority of research is being undertaken among individuals living in ethnic enclaves due to the lack of an ethnic or racial identifier that may help identify Arab Americans from population-based studies. Studies highlight the conflicting evidence in the prevalence of diabetes and cardiovascular disease based on study sample, an increased understanding of cancer incidence and barriers to identification, and an increased level of knowledge regarding mental health and sexual health needs in the population. Information on health behaviors has also increased, with a better understanding of physical activity, alcohol and drug use, and vaccination.

**Conclusion:** More research on Arab American health is needed to identify risks and needs of this marginalized population given the current social and political climate in the United States, especially with regard to acculturation status and immigrant generation status. We provide recommendations on approaches that may help improve our understanding of Arab American health.

## Introduction

Race and ethnicity are a dominant part of national and academic conversations in the United States. One group that is historically represented but largely absent from national and academic conversations about race and ethnicity are Arab Americans. Arab Americans' classification within the United States' racial schema as White makes them invisible as a minority group. This is at odds with politics, war, and violence in the Middle East and the stigmatizing representation of Arabs in the media. Additionally, a recent report by the Arab American Institute Foundation has found that in line with the general surge of bias-motivated violence in the past few years, Arab Americans are at increased risk for hate crimes (1). This discrepancy between having a highly visible and stigmatized group that also has a general lack of visibility in official documents or health records places Arab Americans in an unusual position in the American healthcare system—they are a group that both exists in the public consciousness but is also un-counted and hence has not been a part of a national reckoning with its needs and particularities (2). Aiming to address this gap, we set out to comprehensively review the Arab American health literature, updating a previous review that was conducted a decade ago (3). We examine literature on the health behaviors (actions taken to maintain, attain, or regain good health and prevent illness), health outcomes (an illness, condition, or state that impacts the length or quality of a person's life), and sub-populations within the large Arab American population with particular health needs.

Arab Americans are those individuals with ancestral, cultural, ethnic, linguistic, familial, or heritage ties to one or more of the 22 Arab League countries. It is estimated that there are approximately 3.5 million Arab Americans living in the United States (4). Arabs are disproportionately represented among recent immigrants to the United States due to war and instability in the Middle East. The diversity in country of origin of Arab immigrants makes understanding health indicators difficult, as the differences in health outcomes among Arabic League countries can be disparate. Social determinants in these countries vary dramatically from high poverty and war torn to highly affluent and stable. Arab Americans have varied nativity in the United States: foreign-born (first generation), U.S. born children of immigrants (1.5 and second generation), or U.S. born to U.S. born parents (third and greater generations) (5). The diversity in country of origin, geographic location, tenure in the United States, and acculturation makes studying Arab Americans difficult and the task of generally understanding Arab American health monumental.

A history of Arab American immigration to the United States has been published elsewhere (6). Prior to the 1940's the position of Arab immigrants in the United States' racial system was unclear if not ambiguous (2). The Census Bureau decided in the 1940's that Arab Americans were to be treated like other European immigrant communities (7). The Office of Management and Budget of the United States government has outlined that Arab Americans belong to the “White” racial category, as having origins from the Middle East (8). Arab American's place in society was complicated by developments in the Middle East that led to increased discrimination and exclusion of this population since the 1960's and 1970's (1). Arab American organizations and community members began advocating for a dedicated Middle East and North African (MENA) identifier on the U.S. Census in the 1990's (9). A test of this category was done on the 2015 Census but recently the U.S. Census decided not to include the MENA category on the 2020 Census^1^.

The aim of this review is to provide an updated assessment of the peer-reviewed literature concerned with the health of Arab Americans living in the United States and to summarize key health indicators in this population. A previous review of Arab American health literature published in 2009 reviewed 34 research studies and found little consensus regarding the burden of cardiovascular disease and diabetes in Arab Americans, little information about cancer or mental health disorders, and mixed information regarding whether social determinants of health differed for Arab Americans when compared to other populations (3). We aim to update this review with information on more recently published studies in this population and to synthesize current knowledge about health risks facing Arab Americans to better inform interventions for this vulnerable population and to encourage additional research.

## Methods

The main aim of this study is to summarize the state of the medical and public health literature describing the mental and physical health outcomes and needs of Arab Americans published prior to January 1, 2017. We conducted a comprehensive review following many of the reporting guidelines and criteria set forth by the Preferred Reporting Items of Systematic Reviews (PRISMA) (10). A search was conducted using PubMed/Medline, BIOSYS Previews, CINAHL, Cochrane Reviews, and Web of Science on Arab Americans (specified by a combination of terms including country of origin and regional identifiers) and health related outcomes including tobacco use, cardiovascular disease, stroke, cancer, diabetes, maternal and child health, depression, mental health, trauma, substance abuse and general mental and physical health terms (Supplemental Material). These health behaviors and outcome categories were chosen based on the outcomes most prevalently represented in the previous review and on the authors' expertise. For inclusion, articles needed to describe a study in a population of Arab Americans in the United States. Studies also needed to include a health outcome (defined broadly as physical or mental well-being), be written in English, and be published before January 1, 2017. Qualitative and quantitative research studies were included. Endnote (X8) was the bibliographical data management software used for this review.

**Figure 1:**
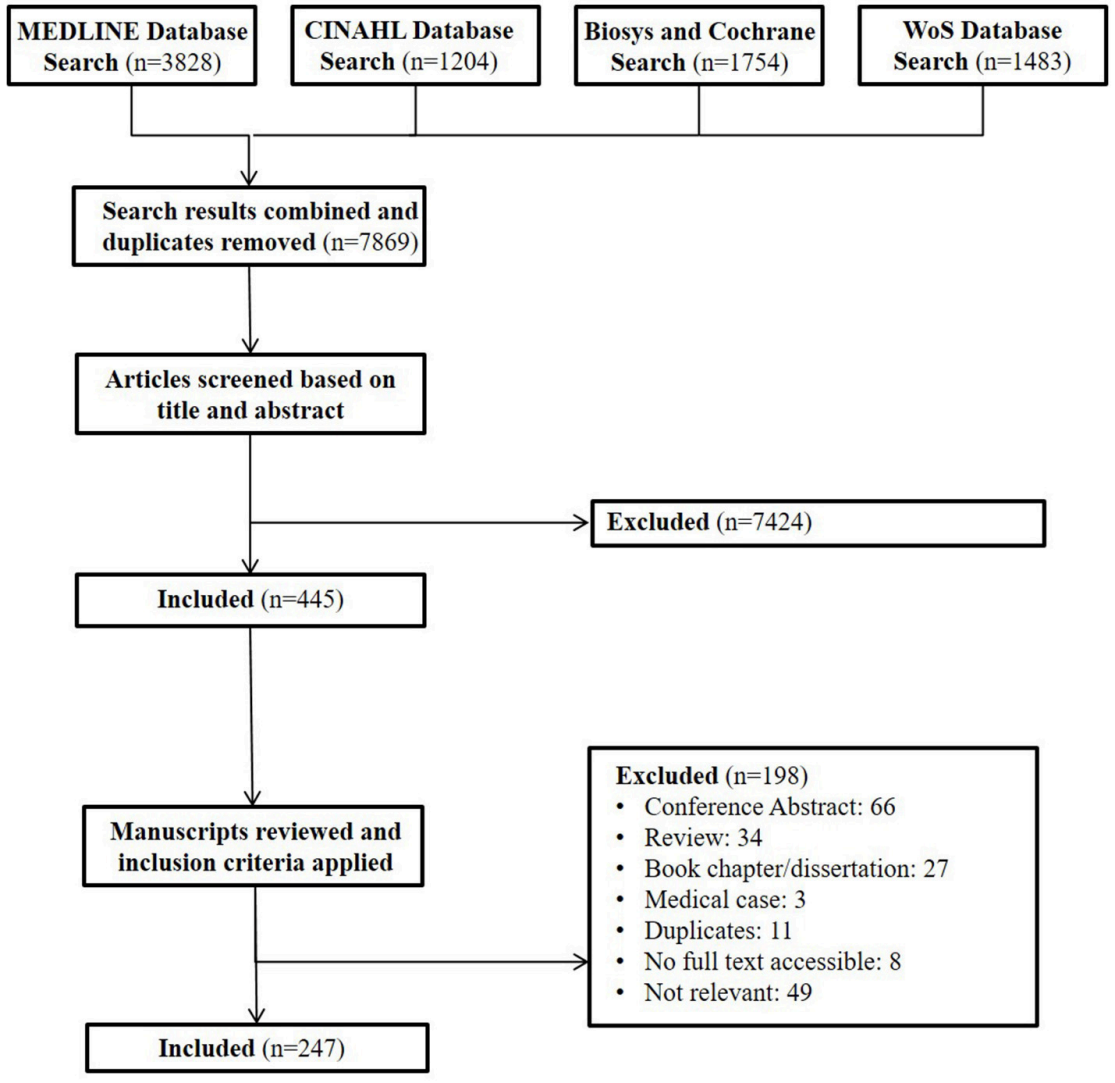
Review cascade for Arab American health. This cascade describes the process through which papers were identified and reviewed for our comprehensive review of Arab American health in the public health and medical literature.

After removing duplicates, each article's title and abstract were read and evaluated by two members of the research team against the inclusion criteria set above. Articles that did not meet the criteria were excluded after conferring with a third research team member. Based on the evaluation process with the titles and abstracts, 7424 articles were removed yielding 445 articles (Figure 1). Full texts of 445 articles were evaluated to ensure each article was a true match to the study criteria. This resulted in the further exclusion of 198 articles, yielding 247 articles in the review. All decisions were agreed upon through review and consensus by at least three research team members (Figure 1).

Epidemiological information on study design, sampling characteristics, study period, geographic origin of samples, and study location were extracted from all papers by multiple investigators. When estimates of prevalence or incidence were reported across studies for the same health outcome, these estimates were recorded as a range. Data points that provided an understanding of the magnitude of illness or disease in Arab Americans were extracted along with notable study designs and observations from qualitative studies. Results are summarized descriptively to help inform readers of the most rigorous, generalizable, and notable takeaways from studies with large sample sizes and rigorous sampling frames (when possible) within health outcomes, behaviors, and vulnerable populations. Quality of data and potential for bias was assessed epidemiologically by more than one investigator and notable exceptions (both positive and negative) are described descriptively within each outcome section of the Results and the Discussion. Due to the heterogeneity in study type and quality of the Arab American literature a systematic quality assessment was not conducted.

## Results

Since the publication of the last review 169 papers were published accounting for 68% of all publications reviewed. We organized our findings around health behaviors, health outcomes, and populations of interest. We examined health behaviors among Arab Americans including tobacco use, physical activity, alcohol and drug use, and vaccination. We examined seven morbidity clusters for health outcomes including diabetes, mental health, women's and child health, cancer, cardiovascular disease, and other health outcomes. Finally, we highlighted two distinct populations of interest for Arab American health: adolescents and the elderly.

Papers describing the health of Somali immigrants and their descendants in the United States (*N* = 66, 27%) accounted for the largest immigrant subgroup, while Iraqi immigrants (*N* = 39, 16%) accounted for the second largest identified subgroup. Nearly half of all papers examined the health of a mix of countries of origin or did not specify the composition of the groups they included (*N* = 122, 49%). The number of United States cities and regions in which Arab American health research is taking place has expanded since the last review. While the majority of studies were still being undertaken in Michigan (*N* = 90, 36%), many other states are represented in the literature including Minnesota (*N* = 30, 12%), California (*N* = 11, 5%), New York (*N* = 8, 3%) and Virginia (*N* = 4, 2%). Eight studies have examined national samples of Arab Americans (3%) while some examined Arab Americans from multiple cities (*N* = 9, 4%).

The epidemiologic composition of publications examined has shifted dramatically since the last review was written. The number of studies testing interventions (*N* = 15, 6%) and the number of longitudinal studies (*N* = 30, 12%) among Arab Americans increased since 2009. Novel methodologies like snowball sampling (*N* = 13, 5%) and web-based surveys (*N* = 3, 1%) were also used in addition to more traditional qualitative methods like focus groups (*N* = 77, 31%). The majority of the literature focused on describing health outcomes in a sample (*N* = 136, 55%) as opposed to examining the relationship between an exposure and outcome.

### Health behaviors

#### Tobacco use

Research regarding tobacco use and addiction among Arab Americans has had a strong foothold and publishing record in Arab American health literature since the early 1990's. The first study on Arab American smoking prevalence occurred in 1992 and aimed to estimate community prevalence among Arab Americans (38.9%) in Michigan (11). Since that first study was published, a number of other studies have examined the prevalence of smoking among Arab American populations, primarily in the Michigan area (12, 13), but also in Houston, Minnesota, and Virginia (14–16) with smoking prevalence ranging from 6 to 45%. Generally, it has been found that less assimilated Arab Americans had a higher dependence on nicotine and tobacco products with the use of tobacco correlating negatively with time spent in the United States (17). More recent literature aims to understand the predictors of water-pipe smoking and of quitting smoking in Arab American populations. Generally, very little evidence was found that Arab Americans desire to quit water-pipe smoking (14). An intervention study performed in a Midwest school aimed at discouraging high school students from starting smoking or continuing smoking showed improvements in non-Arab American and Arab American teens (18).

#### Physical activity

Most of the studies on physical activity were from small convenience samples often performed at religious sites or from religious communities. Further, many of these samples were taken from ethnic enclaves and areas where a large number of Arab immigrants live. Some studies have examined the impact of acculturation on physical activity and found that individuals with lower American acculturation tended to have less physical activity (19) while those found to be more acculturated participated in more physical activities (20). Further, some barriers to physical activity have been identified including a lack of knowledge about how to use machines at the gym (21), lack of money for gym memberships (22), and mixed cultural messaging on body image (19).

#### Alcohol and drug use

A study aimed at estimating the prevalence of binge drinking among Arab Americans at both national and state levels found that nationally Arab Americans had lower prevalence of lifetime alcohol consumption and consumption in the last month than non-Hispanic Whites (23). The national data also showed that Arab American men were 1.78 times more likely to have had alcohol in their lifetime than women (23). An exploratory qualitative study performed by the same investigators aimed to understand the potential reasons for this discrepancy found that alcohol was easily accessible in Arab American communities and environments in Michigan, that social pressures encouraged people to drink at social gatherings, and that populations that were more highly acculturated tended to have higher drinking prevalence than those who were not (24). Some research has been done to understand the low representation of Arab Americans in substance abuse treatment and the presence of language and cultural barriers to Arab Americans enrolling in these programs was uncovered (25).

#### Vaccination

Research on vaccination behavior among Arab Americans lags behind our knowledge of vaccination behavior in other immigrant and minority groups (26–28). A study with data from a national health survey found that Arab Americans had lower estimated rates of recommended vaccinations (flu and pneumonia) when compared to non-Hispanic Whites (29). Only one study among Somali adolescents in Minnesota examined the uptake of human papilloma virus vaccinations. This study showed that while Somali adolescents were accepting of the vaccine, they were less likely to complete the vaccine series when compared to non-Arab, non-Hispanic Whites in the same area (30).

### Morbidity clusters

#### Diabetes

Estimates of diabetes prevalence in Arab American populations range from 4.8 to 23% (31–33). Due to the high prevalence of diabetes and higher odds of diabetes when compared to non-Arab, non-Hispanic Whites found in many Arab American populations, including Somali Americans (34), researchers have examined biological and genetic pathways for these differences. Specifically, work has found that vitamin D insufficiency and hypovitaminosis D were common among Arab Americans and were linked to insulin resistance, metabolic syndrome and glucose intolerance in Arab American men (35). Researchers have also hypothesized that differences in disease burden may also be a result of lack of knowledge and care in this marginalized population.

A number of studies found a lack of appropriate diabetes education tools for Arab Americans (36, 37). Cultural and linguistic deficiencies in the existing educational literature may be preventing Arab Americans from fully understanding their risk for diabetes and their ability to improve their health. Cost and access to appropriate healthcare were barriers identified in a number of studies (38). Acculturation was found to influence diabetes control differently for men than for women. Negative associations between Arab acculturation and diabetes risk were found among Arab American men while American acculturation was found to be associated with diabetes risk among women (39). There were some religious barriers to diabetes control. Some woman cited concerns about modesty that prevented them from exercising properly (36). Ramadan poses a potential barrier to diabetes control in this population as well (40).

One of the first intervention studies implemented among Arab Americans was the adapted Diabetes Prevention Program (DPP) group lifestyle intervention (41, 42). The DPP was tailored linguistically and culturally to Arab American populations in the Dearborn Michigan area to encourage individuals to lose weight and understand their risk for diabetes. The program was successful, with individuals losing weight if they had family support and were appropriately educated on the benefits of the program. This intervention was one of the first successful implementations of intervention research in the Arab American community. The authors attribute their success to targeting gaps in knowledge and reducing misconceptions in recruiting and promoting participation. Arab Americans who perceived they were at increased risk of diabetes were more willing to participate in a lifestyle intervention than those who did not perceive they were at risk (43).

#### Mental health

Research on mental health outcomes and the needs of Arab Americans with regard to mental health has increased since September 11th. While discrimination against Arab Americans is not new, discrimination, and stigmatization have increased in the United States over the past two decades (1, 44). Additionally, many Arab immigrants come to the United States from war torn and conflict-ridden regions increasing their exposure to traumatic and stressful experiences. Studies have documented higher incidence of psychological distress in the years following September 11th among Arab Americans (45). The overall prevalence of depression and other adverse mental health outcomes in Arab Americans is still relatively unknown. Recent studies among Arab American adolescents estimate that 14% of Arab Americans living in the Dearborn ethnic enclave were diagnosed with depression (46), while another online study found that 50% of Arab-American respondents met criteria for depression (44). In particular, studies among Iraqi refugees suggested that this group was at particularly high risk for PTSD and overall health problems (47) and that a longer tenure in the United States was associated with increased depression for this population (48). The effort to estimate this psychological burden is deterred by a number of methodological challenges including the inability to easily identify Arab populations (49), the negative cultural attitudes toward counseling and psychotherapy (50–54), and the lack of targeted and culturally competent mental health services (52, 55) for Arab Americans.

Anti-Arab sentiment was associated with poor mental health outcomes including depression, distress, and unhappiness in Arab American populations (45, 56). Stress, specifically related to migration and the immigration experience, was examined among a group of Arab immigrant woman and found that post-immigration related stressors were correlated with depression and PTSD (57). In a study of Iraqi immigrants, pre-migration trauma was associated with higher rates of depression and PTSD (58). In one of a few randomized clinical trials reviewed, Iraqi refugees who had experienced post-traumatic stress were randomized to brief narrative exposure therapy or control (59). Investigators found that those who received brief narrative exposure therapy had greater posttraumatic growth and increased well-being alongside reduced symptomology for depression (59). Evidence suggests that there were challenges to psychotherapy for Arab Americans including hesitancy in discussing family problems outside of the family and a hesitancy to seek treatment. When individuals sought therapy, therapists reported a large amount of intergenerational conflicts and a difficulty to adjust to mainstream culture as primary complaints (50).

#### Women's and child health

The research in Arab American women's and child health is primarily focused on intimate partner violence, sexual health, obstetrics, and birth outcomes.

##### Intimate partner violence

Some studies found high prevalence of intimate partner violence in Arab American communities (60). Generally, Arab American woman exposed to intimate partner violence were at higher risk of depression (61). Woman who experienced intimate partner violence had many barriers to receiving care and support including language barriers, fear associated with discrimination, lack of culturally sensitive help, and a lack of trust of American providers (60, 62). There was mixed evidence for police intervention and the use of shelters for Arab American women (62). Some work has been done to understand the structural causes of intimate partner violence in Arab American populations including dependence on male relationships for stability and safety post immigration (62), patriarchal Arab culture, the lack of cultural support for seeking marital help outside of the family (61), and family honor, and blaming (62, 63). There is generally a critical need for domestic violence awareness and prevention programs in Arab American populations (64). Additionally, educating women about their rights and the care available to them may improve health seeking behavior alongside the increase in culturally competent health care providers for Arab American patients (60, 62, 63).

##### Female genital mutilation and sexual health

The majority of the literature regarding the sexual health care needs of Arab American women focused on Somali women and those who experienced female genital mutilation. Female genital mutilation is the practice of partially or totally removing external genitalia of young girls and woman for nonmedical reasons (65). This practice is common in 30 countries in Africa, the Middle East and Asia (65). The prevalence and practice of female genital mutilation practices in the United States was low and rare (66, 67), but the number of those who come to the United States having experienced this in other countries was high (66). More research is needed in more diverse populations to understand the FGM practices in the United States and their impact on Arab American women's sexual health.

Knowledge and care for sexually transmitted diseases and HIV among Arab Americans was relatively low. While few research studies examined the sexual health needs of Arab American women, a study performed among Somali immigrants found low knowledge of STI/HIV risk factors, low condom use, and low incidence of extra-marital sex (66).

##### Obstetrics

All studies aiming to understand the obstetric needs of Arab Americans focused on Somali American populations. Among Somali refugees, women preferred little obstetric intervention (68) and clinicians who were more conservative with the use of C-sections (69, 70). Some authors noted that this was due to the fact that many women felt that in Somalia death after obstetric intervention was common (68) and many believed that there was a risk of not having future children after intervention (71). Prenatal education and programs helping mothers understand the options for obstetric care and reducing language barriers between patients and providers could reduce adverse obstetric outcomes (72–74).

##### Birth

Arab immigrants had lower odds of pre-term birth than U.S.-born mothers (75). This trend is not unique to Arab Americans but to many immigrant groups (76). Some theorize that the reasons for this difference in pre-term birth includes the fact that immigrants who are able to immigrate to the United States are those that are healthy (77). Arab mothers tended to be healthier than non-Arab mothers with less tobacco use (78), less pregnancy-related hypertension and diabetes (79), lower rates of birth defects (80), and more consistent prenatal care (81). Somali women in particular faced poor obstetric outcomes due to poorer prenatal care (70) and feeling vulnerable and uninformed, but their outcomes improved with doula support (82). While there is great interest in whether or not adverse birth outcomes increased after September 11th, (83) did not find evidence of increased adverse birth outcomes after September 11th despite showing that stress and discrimination increase this risk among Arab mothers (84).

#### Cancer

Cancer rates among Arab American populations are relatively unknown, although the majority of work in this field has focused on breast cancer.

##### Breast cancer

Whether or not Arab Americans have a higher breast cancer associated mortality rate than non-Arab, non-Hispanic Whites is unclear. Evidence was found in both directions (85, 86). Arab American women tend to be diagnosed at a later stage of breast cancer and yet had higher overall survival than European and American women (87, 88). Barriers to breast cancer screening for Arab Americans included immigration-related barriers, fear, lack of knowledge, and access issues (89–94). Arab American women with higher levels of education and who have lived in the United States for long periods of time were more likely to get screened for breast cancer than their counterparts (95). Fatalism and religious beliefs have been cited by some authors as barriers to breast cancer screening (96). A recent study by Padela et al. found no correlation between fatalism and screening rates among Arab Americans (95).

##### Other cancer

Studies examining the perceptions of cancer among Arab Americans found that education, employment status, and the length of time one spends in the United States affected knowledge of cancer and screening (97). Many Arab Americans generally feared cancer (98) and some believed in keeping cancer diagnoses a secret in order to protect the image of the family in social settings (99). In one study of Arab American men and women, women were found to be more knowledgeable about the modifiable risk factors associated with cancer but that changing food habits was a point of concern (98). To improve perceptions and knowledge about cancer in Arab American populations, community educational programs targeting practices and knowledge within the Arab American community may help create change and awareness of risks (97, 99).

The barriers to screening and treatment of cancer among Arab Americans included lack of knowledge (100–103), religious and cultural beliefs about sickness (100, 104), fear and embarrassment (98, 103), language (101, 104), lack of culturally sensitive healthcare providers (100), lack of access to healthcare (105), and a need to maintain secrecy of sickness or disease (99). Community based participatory research was conducted among Arab Americans to address cancer education, prevention, and screening (106). This type of research was successful and found to increase cancer screening rates among an Arab American community in Detroit and can be used in future studies aiming to improve education and screening of other diseases.

Studies which include Arab Americans that aimed to estimate the incidence of particular cancers primarily focused on two comparisons: comparisons within Arab Americans (place of birth, gender, and country of origin) and comparing Arab American incidence to non-Hispanic, non-Arab White cancer incidence. Differences were observed in the incidence of cancer between foreign-born and U.S.-born Arab Americans and also between male and female Arab Americans (107). Differences of cancer incidence by country of origin were also found (108). Arab Americans, especially women, were at increased risk for thyroid cancer (107–110). Arab American men were found to be at increased risk of lung and prostate cancer when compared to Hispanic men, but had lower rates when compared to Black men (110). Manifestations of cancer also differed with Arab American men suffering from higher urinary incontinence associated with prostate cancer than White men (111). Arab American men had higher rates of bladder cancer than both Hispanic and black men (110). Risk factors for cancers differed between Arab American and non-Hispanic, non-Arab White women. White women tended to have a higher incidence of hormone, tobacco, and alcohol use while Arab American women had high vitamin D related deficiency and radiation exposure (109).

#### Cardiovascular disease

There is a surprising lack of reliable and nationally representative data on cardiovascular disease among Arab Americans. Despite the fact that Arab Americans are generally cited to have higher cardiovascular disease risk than the general population, there is little evidence for this from the existing literature.

Prevalence of self-reported hypertension among Arab Americans (13.4%) was found to be lower than prevalence among non-Hispanic Whites (24.5%) in an analysis of the 2000–2003 National Health Interview Survey (33). A cross-sectional descriptive study among Arab Americans in Southern California found a high prevalence of hypertension (36.5 and 39.7% pre-hypertensive) (112).

A cross-sectional study among Arab Americans in Michigan found an overall prevalence of self-reported heart disease of 7.1% and that Arab Americans were four times more likely to have heart disease than Black Americans in this sample (113). Studies of hypercholesterolemia found prevalence ranged from 24.6 to 44.8% among Arab Americans in various national and community-level convenience samples (39, 114). These numbers were well below the national average of 50.4% hypercholesterolemia in the United States population, but were not rigorously estimated using national and representative sampling methods (114).

Tailakh et al. attempted to understand the awareness of hypertension among a cross-sectional sample of Arab Americans in Southern California and determined that only 67.4% were aware of their hypertension and 52.2% were taking their antihypertensive medication (with 46% of those on medication having controlled blood pressure) (112). To our knowledge, no interventions have been tested to alter cardiovascular status among Arab Americans or influence the risk of developing cardiovascular disease in this population. This is in contrast to the numerous studies developing interventions to improve cardiovascular disease in the United States. While there is no mechanistic reason why interventions developed in other communities would not be transferrable to Arab Americans, it will be important to develop culturally relevant cardiovascular disease interventions for this population.

#### Other health issues

##### Infectious disease

The majority of research on infectious disease outcomes among Arab Americans focused on Somali immigrant populations in the Minnesota area (115, 116). More recent literature examined Hepatitis C among Somali immigrants in Minnesota and among Arabs in Southeast Michigan. Somali immigrants in Minnesota had high rates of hepatitis and hepatocellular carcinoma suggesting targeted interventions were needed for this population (117). The prevalence of Hepatitis C virus antibodies among Arab Americans in Southeast Michigan was found to be triple the national average (5.4%) suggesting the need for more studies assessing the burden of Hepatitis C in this community (118).

##### Asthma

Asthma prevalence has been found to vary by racial and ethnic groups in the United States (119). Evidence from recent studies shows that Arab Americans tended to have lower prevalence of asthma (9.4%) than other racial and ethnic minorities including non-Hispanic Whites and Black Americans (14.4%) (120). An important initiative, the Arab American Environmental Health Project (AAEHP) aimed to understand the impact of the environment on health among Arab Americans populations. One of the first studies published from this interdisciplinary project suggested that asthma prevalence was higher than previously reported among Arab Americans (16%) but that asthma was more strongly correlated with environmental exposure among those with hypertension (121). Asthma management was also found to vary by English language fluency and acculturation variables, suggesting targeted interventions may need to be put in place to help those Arab Americans affected by asthma (122).

### Populations of interest

While many studies focused attention on the adult Arab American population in the United States, there are at-risk populations within this community that have been explicitly studied.

#### Elderly

Global demographics have shifted such that life expectancies are longer and many countries and populations are experiencing aging. The attention paid to the needs of elderly Arab Americans was minimal suggesting this as a future area of research. Better understanding the healthcare, social, and medical needs of the aging Arab American population in the United States is an important and urgent area of study. In our literature review, only three papers examined the needs of older Arab Americans. While Dallo et al. found a reduced likelihood of reporting a disability among Arab Americans when compared to other groups of color in the United States (123), Ajrouch synthesized information from a face-to-face survey and found that well-being varied with social capital for Arab American elders (124). More work is needed in this area to understand how to best care for aging Arab Americans and ensure their mental and physical well-being.

#### Adolescents

Adolescent Arab Americans are mostly composed of second generation Arab Americans, born in the United States to immigrant parents. In a large sample of Arab American adolescents, Ahmed et al. found that there was a strong relationship between perceived discrimination and poor mental health. Further, adolescents reporting more religious coping, strong ethnic identity, and religious support were found less likely to be psychologically distressed (125). An analysis by Aroian et al. (126) found that the quality of the mother-adolescent relationship was the most important predictor of adolescent behavior and stress (127). Adolescents experienced a great deal of discrimination from teachers, school administrators, and their classmates suggesting the need for more dedicated research on how discrimination impacts adolescents (128). A recent analysis by Munro-Kramer et al. found two distinct subgroups of Arab American adolescents in their sample from the Midwest: those with multiple high risk behaviors and those with minimal risk behaviors and more positive life experiences (129). This analysis of high risk behaviors like sexual activity, tobacco use, and physical activity provides a perspective on the potential health needs of Arab American adolescents. The research on Arab American adolescents and their unique health risks requires further exploration and research.

## Discussion

In this updated comprehensive literature review of Arab American health, we found that the literature describing Arab American health outcomes and needs in the past decade has increased and expanded in geographic representation (Table 1). Publications discussing the health behaviors (especially physical activity, alcohol and drug use, and vaccination) of Arab Americans have increased in frequency since the publication of the literature review in 2009 alongside studies focusing on sexual health and female genital mutilation. Since the publication of the last review, a more substantial number of studies have examined the mental health needs of the Arab American population (44, 46, 48, 50, 51, 56, 195–202). Recent studies have improved upon the past literature by providing estimates of depression and depression symptoms among larger groups of Arab Americans alongside the examination of the risk factors (and resiliency factors) associated with this improved mental health but very few studies report on interventions to reduce mental health outcomes. Studies of cancer among Arab Americans have also increased in number since the last review, with a number of studies identifying incidence of a variety of cancers in Arab Americans (106–110) and another set of studies identifying barriers to screening, care and treatment in this population (97–105, 203). There have been more studies examining the risk of diabetes and cardiovascular disease in Arab Americans since 2009 (34–43, 112, 121, 141, 204–206), and more recent studies have examined successful targeted interventions to reduce risk factors for these diseases in ethnic enclaves (41–43). The implementation of intervention studies in Arab American populations is an improvement to the literature reported since the last review.

**Table 1:** Characteristics of Arab American health studies identified through comprehensive review are summarized by number of studies identified and proportion of all studies identified.

	**Number of studies identified (*N* = 247)**	**Proportion**	**References**
**TIME PERIOD**			
1980–1989	4	1.6%	(130–133)
1990–1999	10	4.0%	(11, 64, 69, 134–140)
2000–2009	80	32.4%	(12, 13, 17, 25, 31–33, 47, 52, 55, 63, 74, 78, 79, 83, 85, 86, 98, 108, 113–116, 122, 124, 127, 141–194)
2010–2017	153	61.9%	(14–16, 18–24, 29, 30, 34–46, 48, 50, 51, 53, 54, 56–62, 66–68, 70–73, 75, 80–82, 84, 87–97, 99–107, 109–112, 117, 118, 120, 121, 123, 125, 126, 128, 129, 195–265)
**COUNTRY OF ORIGIN OF SAMPLE (NOT EXCLUSIVE)**			
Egypt	11	4.5%	(54, 55, 79, 135, 146, 151, 152, 185, 186, 240, 245)
Iraq	39	15.8%	(11, 31, 47, 48, 54, 58–61, 79, 93, 94, 99, 109, 124–126, 143, 151, 152, 171, 172, 180, 183–186, 193–195, 197, 198, 207, 208, 244, 248, 249, 253, 256)
Jordan	8	3.2%	(54, 79, 86, 90, 137, 152, 185, 249)
Kuwait	3	1.2%	(151, 185, 249)
Lebanon	28	11.3%	(11, 31, 36, 54, 55, 61, 79, 99, 102, 109, 124–126, 143, 151, 152, 180, 183–186, 193, 194, 198, 207, 238, 249, 260)
Palestine	15	6.1%	(11, 54, 55, 61, 79, 86, 90, 143, 146, 151, 152, 180, 185, 186, 207)
Saudi Arabia	6	2.4%	(54, 79, 146, 151, 152, 249)
Somalia	66	26.7%	(15, 21, 22, 30, 34, 51, 53, 54, 66–74, 82, 89, 94, 100, 101, 103–105, 115–117, 144, 149, 158, 159, 163, 167, 177, 178, 182, 191, 192, 199, 201, 204, 206, 209, 210, 212, 214, 218, 219, 222, 224, 226, 227, 230–233, 235, 237, 241, 243, 254, 255, 257, 261, 263)
Sudan	9	3.6%	(54, 98, 140, 152, 153, 164, 209, 253, 257)
Syria	11	4.5%	(11, 54, 79, 99, 124, 143, 151, 152, 185, 186, 249)
Yemen	22	8.9%	(11, 31, 36, 61, 79, 102, 125, 135, 139, 143, 146, 151, 175, 180, 185, 186, 194, 198, 207, 238, 249, 260)
Mixed/Unspecified	122	49.4%	(12–14, 16–20, 23, 25, 29, 32, 33, 35, 37–46, 50, 52, 54–57, 62–64, 75, 78, 80, 81, 83, 84, 87, 88, 91, 92, 95–97, 106–108, 110–112, 114, 118, 120–123, 125, 127–134, 136, 138, 141, 145, 147, 148, 150, 154–157, 162, 165, 166, 168–170, 173, 174, 176, 181, 187–190, 193, 196, 200, 202, 203, 205, 211, 213, 215–217, 220, 221, 223, 225, 228, 229, 234, 236, 239, 242, 246, 247, 251, 252, 258, 259, 262, 264, 265)
**UNITED STATES LOCATION (NOT EXHAUSTIVE)**			
California	11	4.5%	(19, 20, 69, 110, 112, 133, 162, 211, 228, 252, 254)
Washington D.C.	2	0.8%	(86, 90)
Illinois	3	1.2%	(95, 264, 265)
Louisiana	2	0.8%	(39, 81)
Maine	3	1.2%	(73, 212, 226)
Massachusetts	4	1.6%	(71, 93, 94, 235)
Michigan	90	36.4%	(11–13, 24, 25, 31, 32, 36–38, 41–43, 45–47, 56–63, 75, 78, 79, 83–85, 87, 92, 97, 99, 102, 106–111, 113, 118, 120–122, 124–127, 136, 139, 142, 145, 147, 154, 156, 157, 160, 161, 165, 166, 168, 174, 176, 179–181, 183, 184, 187, 190, 193, 194, 196, 197, 202, 207, 208, 217, 220, 221, 223, 225, 234, 238, 243, 246, 248, 258)
Minnesota	30	12.2%	(15, 21, 30, 34, 66, 72, 74, 100, 101, 103–105, 115–117, 140, 144, 158, 163, 170, 178, 192, 201, 210, 218, 222, 224, 237, 257, 263)
New Jersey	2	0.8%	(110, 152)
New York	8	3.2%	(52, 68, 152, 167, 185, 213, 215, 245)
Texas	2	0.8%	(14, 229)
Virginia	4	1.6%	(16, 146, 236, 242)
Washington	3	1.2%	(89, 149, 214)
National	8	3.2%	(23, 29, 33, 123, 155, 189, 247, 259)
Multiple Cities	9	3.6%	(44, 48, 54, 55, 88, 91, 96, 153, 216)
**SAMPLING CHARACTERISTICS (NOT EXCLUSIVE)**			
Convenience sample	171	69.2%	(12–14, 16, 17, 20–22, 25, 36, 37, 39–42, 46, 50–55, 57, 59–64, 67–74, 81, 82, 85–87, 89–106, 111–114, 118, 120, 121, 124–126, 128–135, 137, 139–141, 143–146, 148–154, 157–159, 164, 167–173, 175, 177–186, 188, 190–195, 197–204, 207, 209–214, 216, 219, 220, 223, 226–233, 235, 236, 238–241, 243, 245, 248, 249, 251–257, 260, 262–265)
Population based sample	69	27.9%	(11, 12, 15, 23, 29–35, 38, 43–45, 47, 48, 56, 58, 75, 78–80, 83–85, 88, 107, 108, 110, 115–117, 122, 123, 136, 138, 142, 147, 155, 156, 160–163, 165, 166, 174, 176, 180, 187–189, 196, 205, 206, 208, 215, 218, 221, 222, 225, 234, 237, 246, 247, 250, 258, 259)
Snowball sampling	13	5.3%	(22, 54, 91, 127, 135, 167, 177, 199, 203, 232, 235, 244, 261)
Web-based sampling	3	1.2%	(54, 55, 177)
**STUDY CHARACTERISTICS (NOT EXCLUSIVE)**			
Cross-sectional	196	79.4%	(11–25, 32–40, 43–46, 48, 50–57, 60–64, 66, 71, 72, 74, 81, 82, 86, 89, 91, 93–96, 98–100, 102, 103, 109–114, 117, 118, 120–122, 124, 125, 127–161, 163, 164, 166–195, 197–202, 204–211, 213, 214, 216, 217, 219–221, 223–225, 227–233, 235, 237–242, 244–246, 249, 251–263, 265)
Longitudinal	30	12.2%	(29, 30, 42, 59, 75, 78–80, 83, 84, 88, 92, 105–108, 115, 116, 123, 126, 162, 165, 196, 217, 218, 234, 236, 247, 248, 250)
Intervention	15	6.1%	(18, 41, 42, 46, 59, 87, 92, 97, 104, 106, 150, 215, 223, 236, 243)
Focus groups or interviews	77	31.2%	(19, 21, 24, 36–38, 51–53, 62–64, 66–71, 73, 74, 81, 82, 89, 90, 93, 94, 96, 98–101, 103, 126, 128–130, 136, 137, 139, 143, 144, 148, 158, 159, 167, 169, 171, 178, 185, 191, 192, 195, 199, 201, 207, 210, 212, 214, 215, 219, 224, 227, 229–233, 235, 241, 242, 248, 253, 254, 256, 257, 263, 264)

### Methodological limitations in the literature

This review highlighted some methodological shortcomings of the existing literature on Arab American health that may prevent comparisons of health indicators to other ethnic groups in the United States. First is our inability to generalize findings from ethnic enclaves to areas where Arab Americans may be dispersed among other ethnic populations. The majority of studies aiming to understand health outcomes and health needs of Arab Americans took place within ethnic enclaves, primarily Dearborn Michigan, and Somali enclaves within Minnesota. In fact, regionally representative data sets using rigorous sampling methodologies (that have been invaluable for our understanding of Arab American health needs) have been overused and overpublished in the literature, suggesting that much of the information we have on Arab populations stems from a few datasets (ex. Detroit Arab American 2003 survey) within ethnic enclaves. While the identification and recruitment of Arab individuals in this area is made easier by social and cultural community connections, the lived experiences of these Arabs likely differs from those living in other parts of the country. Further, the large diversity of Arab populations residing in the United States with regard to country of origin, immigrant generation, and religion necessitates the study of diverse and nationally representative Arab samples in future studies.

Second, almost all studies were recruited through convenience sampling and very few studies used rigorous sampling methodologies to recruit samples of Arab Americans. This is in large part due to the absence of an easy way to identify Arab Americans on a wider scale and is a previously mentioned concern by other authors (3, 266). Investigators have used a variety of methods to isolate Arab Americans from larger datasets including Arab surname algorithms, using place of birth and Arabic language as indicators. Little is known about the generalizability of the findings from each of these methods.

Third, there were a general lack of prospective and longitudinal studies performed in this population- most studies were cross-sectional in nature. Longitudinal studies are able to follow individuals for longer periods of time to understand disease development. The longitudinal studies that were performed in this population have aimed to increase education, screening, or knowledge. Very few aim to alter disease status or examine interventions specific to disease reduction. The United States public health community has a long history of successful longitudinal studies aimed at understanding the burden of chronic diseases within particular populations (ex. Framingham Heart Study, Nurses' Health Study etc.) but the richness of these datasets cannot be used to understand Arab American health because, despite their likely presence in these datasets, Arab Americans cannot be isolated. These methodological challenges should be considered in the context of the future research directions recommended.

Unfortunately, many of the methodological shortcomings in the literature have persisted since the publication of the last review (3), suggesting the need for more active collaboration among researchers and institutions to overcome these limitations. These methodological shortcomings made a rigorous quality assessment of the literature difficult to conduct due to heterogeneity in the rigor of studies and the relatively recent nascence of the Arab American health literature. In the absence of a systematic quality assessment of the literature, our literature review could not be considered systematic, a limitation of our work.

While progress has been made in expanding the geographic representation of studies and the inclusion of a few randomized trials, more longitudinal studies with population-representative samples will help improve our understanding of Arab Americans.

### Future research directions

Future studies should aim to understand the diverse healthcare needs of Arab Americans and to better understand how to improve their experiences in healthcare. All institutions and organizations with an interest in helping improve minority health should identify Arab American populations in their databases and provide culturally competent services for improved care. Information taken on intake forms should include expanded information on race, religion, and cultural practices that may inform the care or health decision making of minority populations. Community based participatory research can be used by these institutions to develop interventions and to understand the health needs and desires of Arab American populations alongside the cultural nuances that influence health.

Second, understanding the needs of vulnerable sub-populations within the Arab American community should be prioritized. Understanding health needs within elderly Arab populations and the impact that increased life expectancy will have on these populations is important to our understanding of Arab American health more generally. The cultural processes and traditions associated with elder care and medical care may influence the health needs and resources available to this vulnerable populations over time. Another vulnerable population in need of active study are adolescents, and particularly adolescent women. Due to the compounding issues of acculturation, stress, and puberty, the health behaviors of this population should be better understood to develop targeted interventions. Studies aiming to examine Arab American sexual health should expand beyond the realm of female genital mutilation and attempt to provide culturally competent care and culturally relevant solutions to sexual health issues in this population.

Third, increasing the amount of information collected about acculturation status including immigration generation is important and necessary. Understanding the factors that make Arab American and Arab immigrants resilient will help contextualize Arab American health in the minority health landscape in the United States. Collecting data on religion should become a regular part of studies on mental health among Arab Americans. Evidence shows that assimilated Muslim Arab Americans experience higher levels of discrimination compared to their less assimilated counterparts and assimilated Christian Arab Americans (54). A better understanding of the role of religion could lead to productive collaborations between mental health providers and faith based institutions.

Finally, more creative studies using natural, political, or environmental experiments will also help the medical community understand the needs of this population in relation to the changing social and political environment in the United States. Using novel data streams to more rigorously sample Arab Americans nationally will help the medical community understand the contexts in which Arab Americans are accessing healthcare and may help improve their social experience in the United States overall. Ensuring the security and confidentiality of Arab Americans in these studies should be prioritized and emphasized to continue increasing the confidence of this community with medical and public health research.

### Conclusion

Arab Americans are a socially and politically important immigrant group in the United States that has been increasing in size due to increased immigration over the past decade. More attention is needed to better understand the social and health needs of this population in the context of the changing political climate. Public health and medical researchers can improve their understanding of the health needs of Arab Americans through the identification and active recruitment of Arab Americans, the focus on vulnerable sub-populations, collecting data on acculturation and religion, and using novel longitudinal data streams. Understanding Arab American health needs in the context of the existing racial and minority health landscape in the United States will be important to better understanding immigrant health in the United States.

## Author contributions

NA, AE-S, and SG contributed to the design of the research. NA implemented the research and analyzed the results. NA, AE-S, and SG contributed to the writing of the manuscript.

### Conflict of interest statement

The authors declare that the research was conducted in the absence of any commercial or financial relationships that could be construed as a potential conflict of interest.

## Footnotes

^1^Using Two Separate Questions for Race and Ethnicity in 2018 End-to-End Census: Hearing before the 2020 Census Program Memorandum Series (February 2, 2018, 2018).
