# Supplementary material for: The Health of Arab Americans in the United States: An Updated Comprehensive Literature Review
Source: Front Public Health. 2018 Sep 11;6:262. doi: 10.3389/fpubh.2018.00262 (PMC6141804; doi:10.3389/fpubh.2018.00262)
Supplement: Supplementary file 1 [file Table_1.DOCX]

**Supplemental Information:** *Search terms and strategy for the systematic review and search of Arab American health manuscripts.*

The following search terms and strategy was used in each of the databases examined:

1. (Arab or "Arab American" or "Middle*Eastern" or Algeria* or Bahrain* or Egypt* or Palest* or Iraq* or Leban* or Libya* or Jordan* or Kuwait* or Morocc* or Mauritania* or Oman* or Qatar* or Saudi or "Saudi Arabian" or Somali* or Sudan* or Syria* or Tunisia* or Emirat* or Yemen* or Djibouti or Copt* or Chaldean or Assyrian)
2. (America* or "United States")
3. (health or wellness or disease or disorder or deficiency or illness or "risk factor" or issue or sickness or "ill health" or infection or inflammation or syndrome or condition or infirmity or complaint or tobacco or smok* or water*pipe or "cardiovascular disease" or "heart disease" or infarct* or heart or MI or CVD or atherosclero* or stroke or ischemi* or cancer or neoplas* or tumor or leukemia or lymphoma or diabet* or glucose or metaboli* or matern* or pergnan* or ped* or birth or abuse or depress* or anxiety or PTSD or post*trauma* or substance or mood or schizophren* or mental or psych*)
4. (human* OR homo sapiens OR Man OR Men OR Woman OR Women)
5. (veteran* or military or enlisted)

1 AND 2 AND 3 AND 4 NOT 5
